# Sleep Disorders: A Blind Spot in Disease Burden Research

**DOI:** 10.3389/ijph.2026.1609757

**Published:** 2026-05-06

**Authors:** Luisa Sophie Welter, Christoph Nissen, Björn Rasch, Raphael Heinzer, Alexandre Nikolaus Datta, Ester Piovesana, Martine Bouvier Gallacchi, Virginie Sterpenich, Claudio Lino Alberto Bassetti

**Affiliations:** 1 European Sleep Foundation, Lugano, Switzerland; 2 Swiss Brain Health Foundation, Riazzino, Switzerland; 3 Department of Psychiatry, Faculty of Medicine, University of Geneva, Geneva, Switzerland; 4 Department of Psychiatry, Geneva University Hospital (HUG), Geneva, Switzerland; 5 Division of Cognitive Biopsychology and Methods, Department of Psychology, University of Fribourg, Fribourg, Switzerland; 6 Center for Investigation and Research in Sleep, University Hospital of Lausanne, Lausanne, Switzerland; 7 Department of Pediatric Neurology, Developmental Pediatrics and Neurorehabilitation of Northwest Switzerland, University Children’s Hospital UKBB Basel and Children’s Hospital KSA Aarau, Basel, Switzerland; 8 Department of Neuroscience, Faculty of Medicine, University of Geneva, Geneva, Switzerland; 9 Medical Faculty, University of Bern and Neurology Department, Inselspital, Bern, Switzerland

**Keywords:** disability-adjusted life years, disease burden, global burden of disease, public health, sleep disorders

The Global Burden of Disease (GBD) study is a powerful tool for informing public health strategies and policies. Its comprehensive and regularly updated database enables comparisons across health states, regions, and years. However, certain methodological features, combined with the study’s considerable influence, may inadvertently contribute to the neglect of conditions that are critical to population health. This Editorial highlights the underrepresentation of sleep disorders in current disease burden research, notably the GBD study.

A substantial body of evidence demonstrates that restorative sleep is essential for mental, physical, and societal wellbeing, as well as economic prosperity [1]. Sleep health is now recognized as a major determinant of overall health. For instance, recent research drawing on more than 380,000 participants from the UK Biobank indicates that healthy sleep patterns, such as maintaining a sleep duration of 7–8 h per night, are associated with reduced risks of both overall and site-specific cancers [2]. Concerningly, sleep disturbances are highly prevalent: approximately 25% of the population reports reduced sleep duration (sleep loss) [3], and 10% experience excessive daytime sleepiness [4]. Sleep disorders that reach a clinically significant threshold are also common. For example, severe insomnia affects approximately 8% of the general population [5], and sleep apnea occurs in 9%–38% of adults [6].

Despite this considerable burden, sleep disorders have been absent from GBD publications since the 2004 update [7]. They are currently neither included as primary disorders nor as risk factors [8]. Consequently, key burden metrics such as disability-adjusted life years (DALYs) remain underreported for conditions of inadequate sleep. This gap is notable given that many rare diseases, symptoms, and risk factors are routinely included in GBD analyses. The reason for the exclusion of sleep disorders is unknown to the authors of this Editorial, also considering that GBD estimations could be informed by primary data from large epidemiological resources, such as the UK Biobank [2].

The exclusion of sleep disorders from multi-disease burden reports such as the GBD study has important implications. As these estimates are widely used to derive prevention and healthcare needs, the exclusion of sleep disorders perpetuates their inadequate management across sectors, including prevention, diagnosis, and treatment. This is particularly concerning because improvements in sleep quality and consistency have beneficial spillover effects on mental, neurological, cardiovascular, and metabolic outcomes as well as on overall health [1]. Inadequate sleep also poses a direct threat to population health and safety by increasing the risk of traffic accidents [9]. A recent GBD publication acknowledges that, “Psychosocial factors, such as sleep, stress, and social isolation, are increasingly recognized as contributors to neurological conditions and should be quantified in future analyses.” [10]. While we welcome the inclusion of sleep as a determinant of health, we argue that sleep disorders should also be recognized as primary disorders, as reflected in current disease classification systems, including the DSM-5 and ICD-11.

Through this Editorial, we aim to alert policymakers, clinicians, and researchers to the significant gap in burden data on sleep disorders–a gap that limits evidence-based prevention and healthcare strategies. Incorporating sleep disorders into burden estimation frameworks, such as the GBD study, would enhance disease recognition and represent a critical step toward improving population health.
